# Hemolytic Anemia Caused by Dynamic Left Ventricular Outflow Tract Obstruction Following Surgical Aortic Valve Replacement: A Case Report

**DOI:** 10.7759/cureus.83807

**Published:** 2025-05-09

**Authors:** Hirofumi Haida, Atsuo Mori, Koji Funaishi

**Affiliations:** 1 Cardiovascular Surgery, Kawasaki Municipal Hospital, Kawasaki, JPN

**Keywords:** annular enlargement, aortic valve replacement (avr), hemolysis, left ventricular outflow tract stenosis, morrow procedure, myectomy, surgical myectomy

## Abstract

Hemolysis following surgical aortic valve replacement (SAVR) is typically attributed to paravalvular leakage or prosthetic valve dysfunction. However, we present a rare case of hemolytic anemia caused by dynamic left ventricular outflow tract obstruction (LVOTO) due to septal hypertrophy following SAVR with a bioprosthetic valve. A 78-year-old woman underwent SAVR with annular enlargement via the Manougian procedure. Despite an uneventful intraoperative course and initial recovery, she developed progressive hemolysis postoperatively without evidence of valve dysfunction or paravalvular regurgitation. Echocardiography revealed dynamic LVOTO and moderate mitral regurgitation (MR). Conservative treatment, including beta blockers and calcium channel blockers, led to improvement of the obstruction and resolution of hemolysis. This case highlights the need to consider dynamic LVOTO in the differential diagnosis of post-SAVR hemolysis and to more aggressively evaluate the indication for concomitant septal myectomy in patients with preexisting hypertrophy.

## Introduction

Hemolytic anemia after aortic valve replacement (AVR) is most often caused by paravalvular leakage or prosthetic valve degeneration [1]. In rare cases, hemolytic anemia caused by worsening left ventricular outflow tract obstruction (LVOTO) after AVR has been reported [2,3]. Dynamic LVOTO has been reported in approximately 10% to 15% of patients after surgical aortic valve replacement (SAVR), particularly in individuals with pre-existing septal hypertrophy, small left ventricular (LV) cavity size, and preserved systolic function [4]. Here, we report a unique case of hemolytic anemia caused by postoperative dynamic LVOTO in a patient undergoing SAVR for severe aortic stenosis (AS), managed successfully with pharmacologic therapy. This case underscores the importance of recognizing dynamic LVOTO as a reversible cause of hemolysis and the role of preoperative planning in patients with high-risk features.

## Case presentation

The patient was a 78-year-old woman. She had severe AS, and surgical treatment was indicated. Preoperative examination revealed a calcified lesion in the ascending aortic intima near the sinotubular junction (STJ); therefore, transcatheter aortic valve replacement (TAVR) was considered unsuitable, and SAVR was chosen for this case. Preoperative transthoracic echocardiography (TTE) showed a highly calcified tricuspid aortic valve with a peak velocity of 5.1 m/s and a mean pressure gradient of 65 mmHg. The aortic valve area was 0.67 cm², and a small aortic annulus with a diameter of 17 mm, so we decided to perform a concomitant annular enlargement. A small LV cavity, concentric hypertrophy (interventricular septum thickness: 15 mm), and hyperdynamic systolic function (ejection fraction: 77%) were noted. Mild mitral regurgitation (MR) and a peak LVOT velocity of 2.6 m/s were also observed (Video 1). 

**Video 1:** Preoperative transthoracic echocardiography (TTE) Severe aortic stenosis (AS) with small anulus (17 mm); left ventricular outflow tract obstruction (LVOTO) and systolic anterior motion (SAM) with mild mitral regurgitation (MR) are also noted.

Surgery was performed through a median sternotomy, and cardiopulmonary bypass (CPB) was established in the standard manner. Cardiac arrest was achieved by antegrade and retrograde administration of cardioplegia. The aorta was incised in a J-shape 1.5 cm cephalad of the origin of the right coronary artery, and a calcified lesion was found on the posterior wall of the aorta, which was removed. The aortic valve was tricuspid and highly calcified. The aortic valve was excised, and the annulus was measured. Since the smallest size of the bioprosthetic valve did not pass through, the annulus was enlarged by the Manougian method using a Hemashield (Maquet Cardiovascular, San Jose, CA, USA) patch and replaced with a 19 mm Edwards INSPIRIS RESILIA bioprosthesis (Edwards LifeSciences, Irvine, CA, USA). The width of the patch was 8 mm, and the patch was threaded with four stitches of 2-0 thread with a pledget from outside the aorta. Transesophageal echocardiography (TEE) post CPB showed well-seated valve function with no paravalvular regurgitation and normal biventricular function. No additional procedures, such as septal myectomy, were performed, as intraoperative hemodynamics were stable and the LVOT appeared unobstructed.

Postoperative respiratory failure was observed, and the patient required a ventilator until postoperative day (POD) 3. After removing the ventilator, the patient's progress was uneventful. Cardiac rehabilitation was started in the general ward, but elevated LDH and progression of anemia were observed from POD 7. TTE on POD 10 revealed a peak LVOT velocity of 4.7 m/s, a peak gradient of 90 mmHg, and dynamic narrowing of the LVOT during systole. Moderate MR and systolic anterior motion (SAM) of the anterior mitral leaflet were noted (Video 2). The prosthetic aortic valve showed normal leaflet motion and no evidence of regurgitation or obstruction. 

**Video 2:** Transthoracic echocardiography (TTE) at 10 days postoperatively The left ventricular outflow tract obstruction (LVOTO) aggravated, and the mitral regurgitation (MR) deteriorated to moderate. Prosthetic valve function was normal. Paravalvular leak (PVL) was not observed.

**Figure 1:**
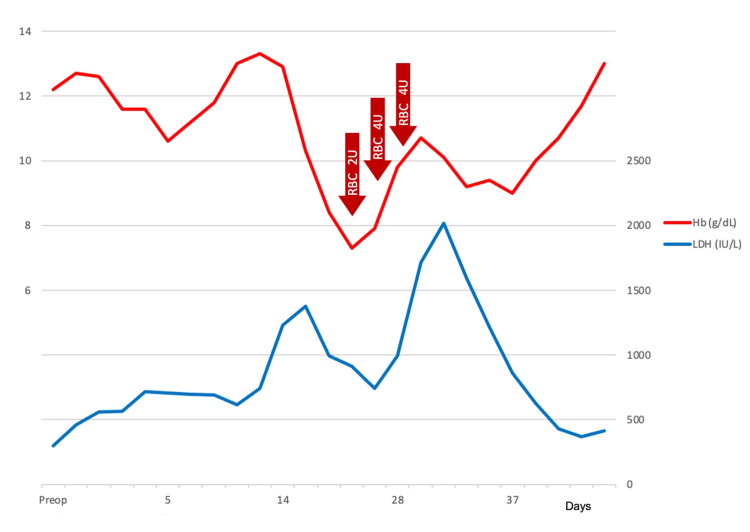
Postoperative hemoglobin (Hb) and lactate dehydrogenase (LDH) trends From postoperative day 7, Hb levels began to decline from 10.5 to 7.3 g/dL, and serum LDH levels increased from 300 to 2020 IU/L. Transfusions of red blood cells (RBCs) were conducted during the course. The hemolysis gradually improved with medication adjustment. Normal ranges: Hb 11.6-14.8 g/dL; LDH 124-222 IU/L

​​​During the course of the treatment, the maximum lactate dehydrogenase (LDH) was elevated to 2020 IU/L, and hemoglobin (Hb) decreased to 7.3 g/dL, requiring blood transfusion (Figure 1). Haptoglobin was undetectable, and indirect bilirubin was elevated. The reticulocyte count was 6.8%. Peripheral blood smear showed fragmented red blood cells. 

Diuretics were discontinued, and beta blocker therapy (bisoprolol 5 mg/day) was initiated. Diltiazem (100 mg/day) was added to further reduce myocardial contractility. Over two weeks, LVOT gradients decreased (peak 2.3 m/s, peak gradient of 10 mmHg), SAM resolved, and MR improved to mild (Video 3). Concurrently, LDH decreased to 427 IU/L, and Hb increased to 10.7 g/dL without further transfusions. The patient was discharged on POD 40.

**Video 3:** Transthoracic echocardiography (TTE) at discharge This TTE was obtained after medication adjustment. Remission of left ventricular outflow tract obstruction (LVOTO) and the regression of mitral regurgitation (MR) to mild were observed.

**Figure 2:**
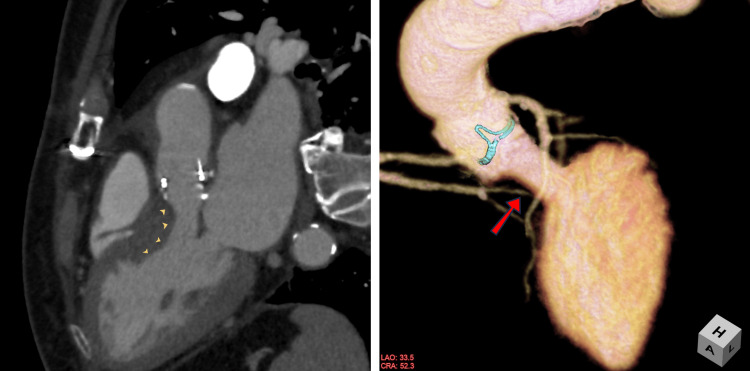
Postoperative CT showed residual ventricular septal muscle (left, arrow head), and narrowed left ventricular outflow tract (right, arrow).

At the three-month follow-up, contrast-enhanced CT scan showed residual left ventricular outflow tract stenosis (Figure 2), and TTE revealed persistent mild LVOTO (peak 2.3 m/s) and mild MR without SAM (Video 4). At six months, LDH was 400 IU/L, Hb was 13.0 g/dL, and the patient remained asymptomatic.

**Video 4:** Transthoracic echocardiography (TTE) at three months postoperatively Prosthetic valve function was normal. Residual left ventricular outflow tract obstruction (LVOTO) and a pressure gradient in the LVOTO area are noted (Vmax 2.3 2.3m/sec, Pmean 8 mmHg).

## Discussion

Hemolytic anemia following AVR is most commonly associated with paravalvular leakage or structural dysfunction of the prosthetic valve [1]. However, LVOTO can also be an underrecognized cause [2,3]. In our patient, hemolysis developed after SAVR despite the absence of prosthetic valve dysfunction or paravalvular leakage. Instead, postoperative echocardiography revealed dynamic LVOTO, which appeared to contribute directly to the hemolysis. Pharmacological intervention, rather than surgical reintervention, led to a resolution of both the LVOTO and the hemolytic findings.

Dynamic LVOTO has been reported in up to 15% of patients after SAVR, particularly among those with small left ventricular cavities, hyperdynamic systolic function, and basal septal hypertrophy [4]. In the present case, preoperative imaging revealed LVOT narrowing due to septal hypertrophy. However, the team proceeded with annular enlargement using the Manouguian procedure without performing myectomy. One reason for this decision was the inherent challenge of accurately evaluating dynamic LVOTO in patients with severe AS, where the high afterload can mask intraventricular gradients [5]. However, this case highlights that even in the absence of overt obstruction preoperatively, structural features suggestive of possible LVOTO should prompt proactive consideration of septal myectomy at the time of SAVR.

After surgery, initial imaging showed no evidence of paravalvular leakage or prosthetic dysfunction. However, from postoperative day seven, the patient developed anemia and markedly elevated LDH, consistent with hemolysis. Echocardiography confirmed dynamic LVOTO with a moderate gradient and associated mitral regurgitation. While LVOTO alone has been reported to cause hemolysis, the gradient in this case was not as severe as in previously published cases [6,7]. We therefore suspect that hemolysis was due not merely to obstruction but to high-velocity jet flow striking artificial surfaces, specifically, the prosthetic valve or the patch material from annular enlargement, leading to mechanical shear stress on red blood cells.

Importantly, no paravalvular leakage was identified, reinforcing that LVOTO alone, particularly when the flow jet is eccentrically directed toward prosthetic components or patch material, may be sufficient to cause clinically significant hemolysis. This emphasizes the need to evaluate not just the presence of obstruction, but also the direction and target of blood flow during and after surgery.

Treatment of dynamic LVOTO centers on reducing hypercontractility and increasing left ventricular volume through beta-blockers, negative inotropes, and volume management [8,9]. In our case, pharmacological therapy with bisoprolol and diltiazem successfully resolved the obstruction and hemolysis. While surgical intervention, including delayed myectomy, may be required in refractory cases, this experience supports medical management as an effective first-line approach when the obstruction is dynamic and not structural.

With the increasing use of TAVI in AS patients, anatomical features such as basal septal hypertrophy remain important considerations [10,11]. The 2020 American College of Cardiology (ACC)/American Heart Association (AHA) guidelines list septal hypertrophy requiring myectomy as a factor favoring SAVR over TAVI [12]. However, SAVR alone does not always resolve subvalvular obstruction. Our experience underscores that when preoperative imaging suggests LVOT narrowing or septal thickening, especially in the presence of a small ventricle, concomitant myectomy should be actively considered, even if dynamic obstruction cannot be fully confirmed before surgery [13]. Annular enlargement alone may not be sufficient to prevent postoperative LVOTO and its complications.

In summary, this case emphasizes the importance of recognizing LVOTO as a potential cause of hemolysis after AVR, even in the absence of significant paravalvular leakage or prosthetic valve dysfunction. It also highlights the need for careful preoperative assessment of septal morphology in AS patients and supports the use of medical therapy as a first-line treatment in dynamic forms of LVOTO. For patients undergoing SAVR with evidence of septal hypertrophy and LVOT narrowing, the addition of a myectomy (e.g., Morrow procedure) should be considered proactively to prevent postoperative complications such as hemolysis.

## Conclusions

We present a rare case of dynamic LVOTO-induced hemolysis following SAVR in a patient with septal hypertrophy. The diagnosis was confirmed by exclusion of common causes and response to medical therapy. This case highlights the importance of preoperative recognition of anatomical risk factors and the need to consider septal myectomy when appropriate. Dynamic LVOTO should be included in the differential diagnosis of post-AVR hemolysis, especially in the absence of prosthetic valve dysfunction.
